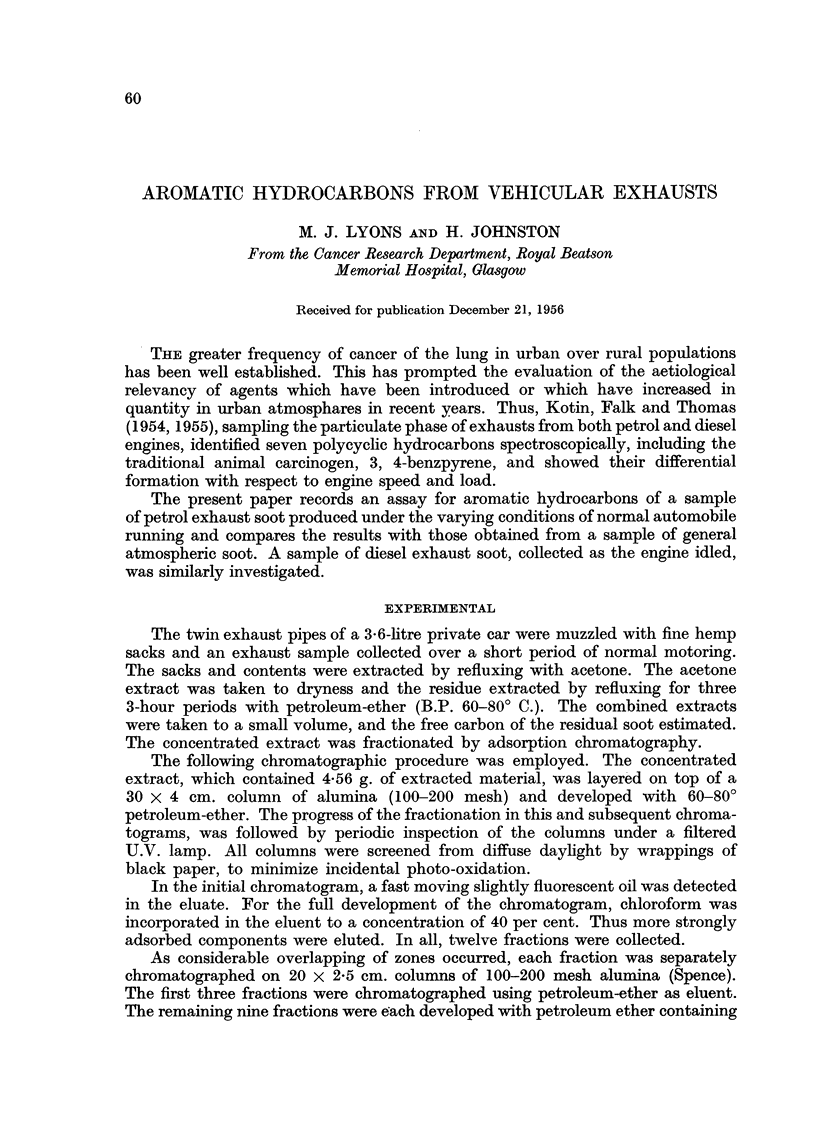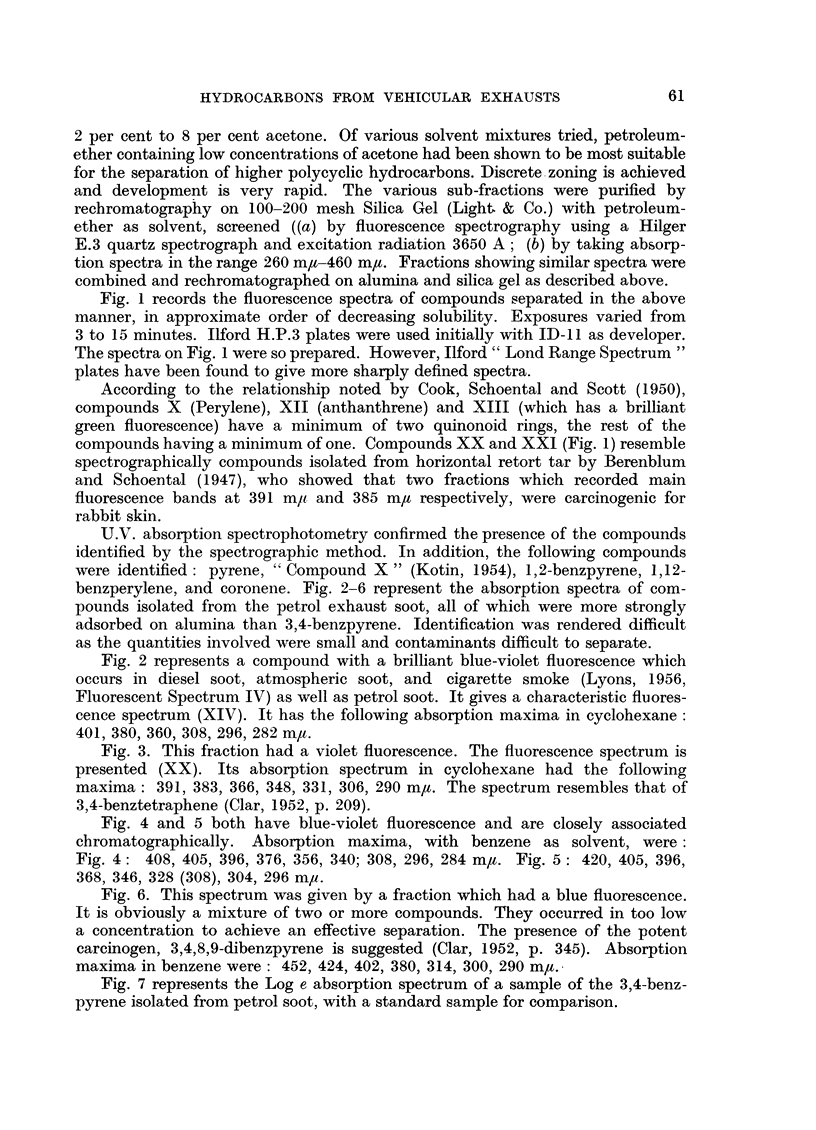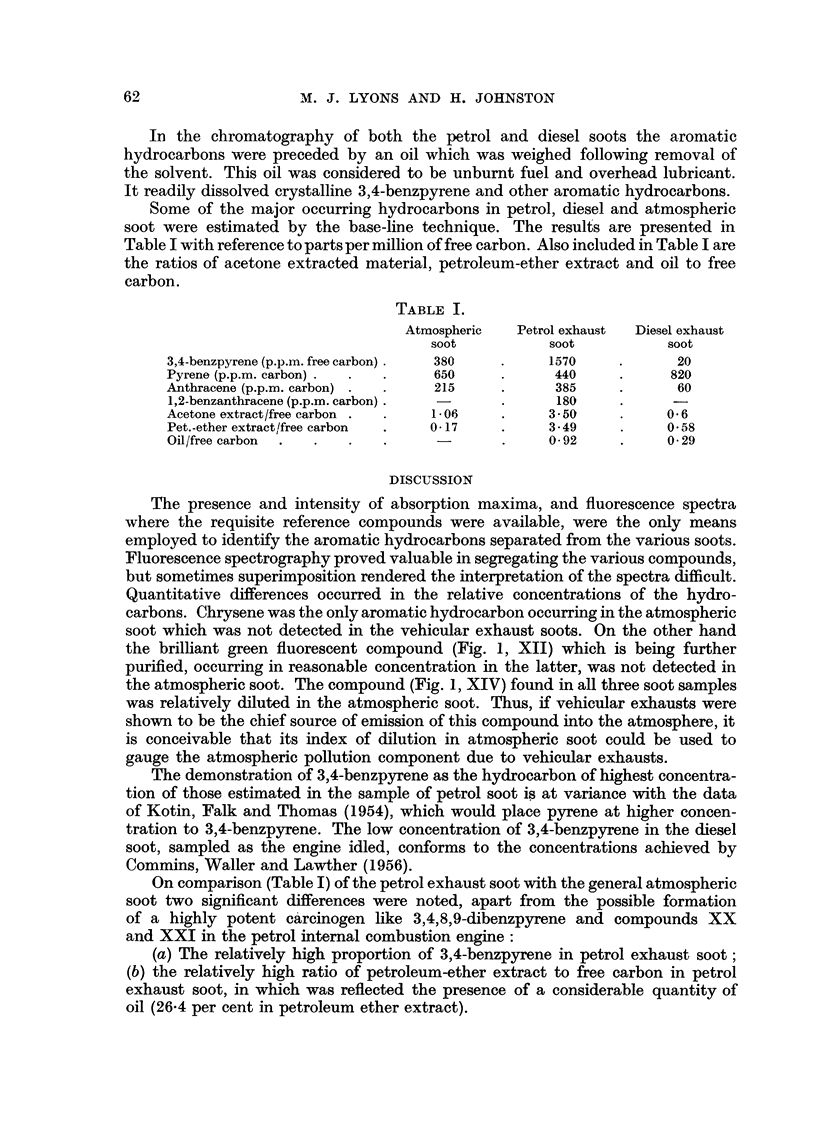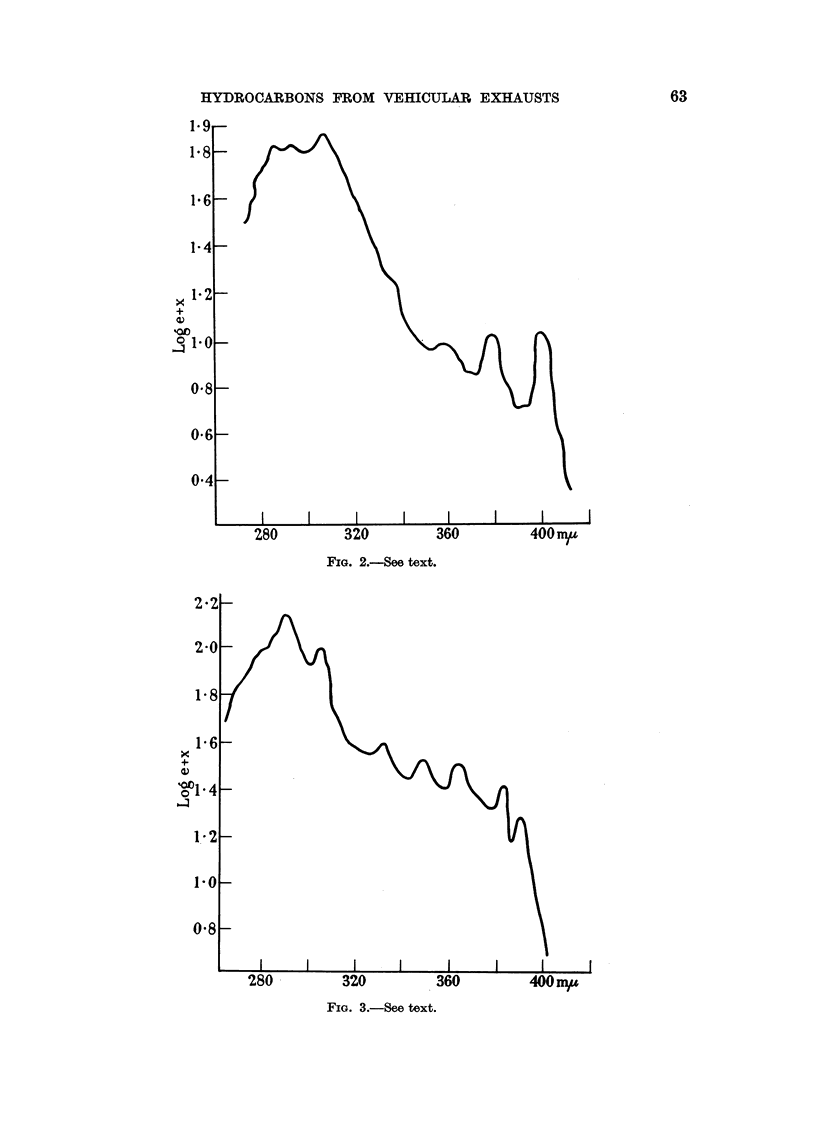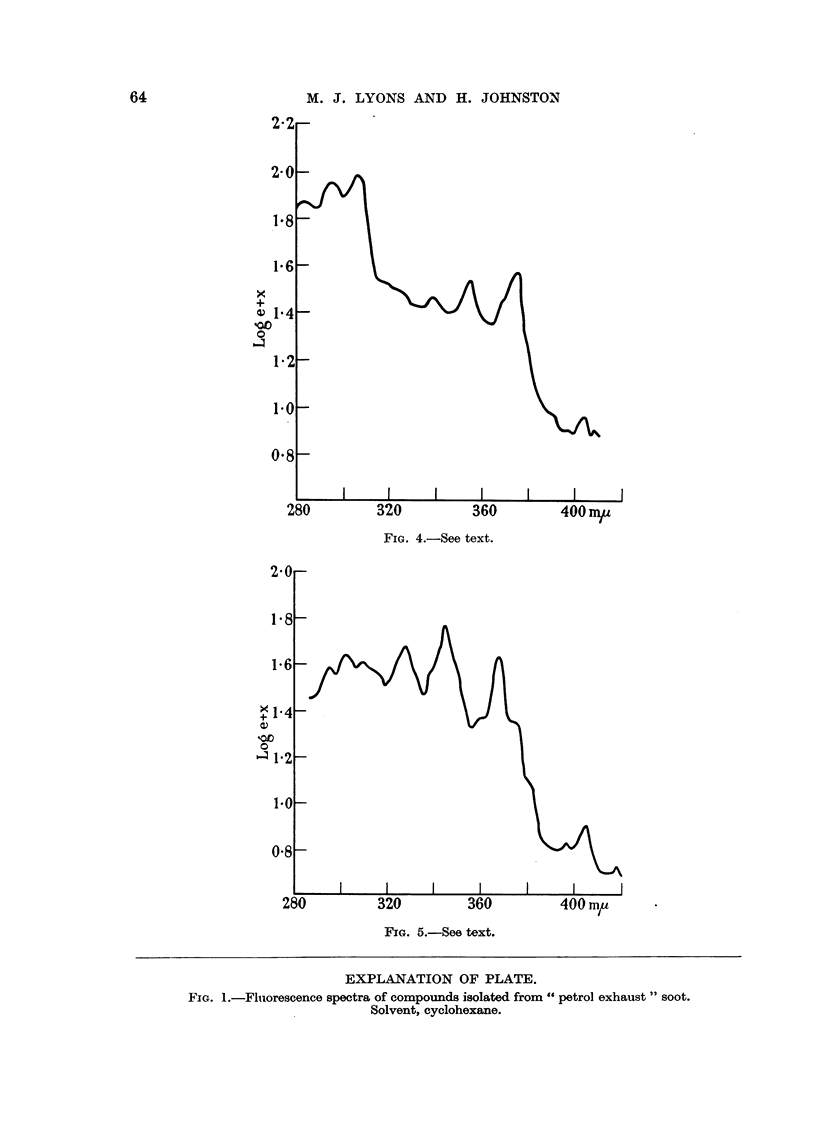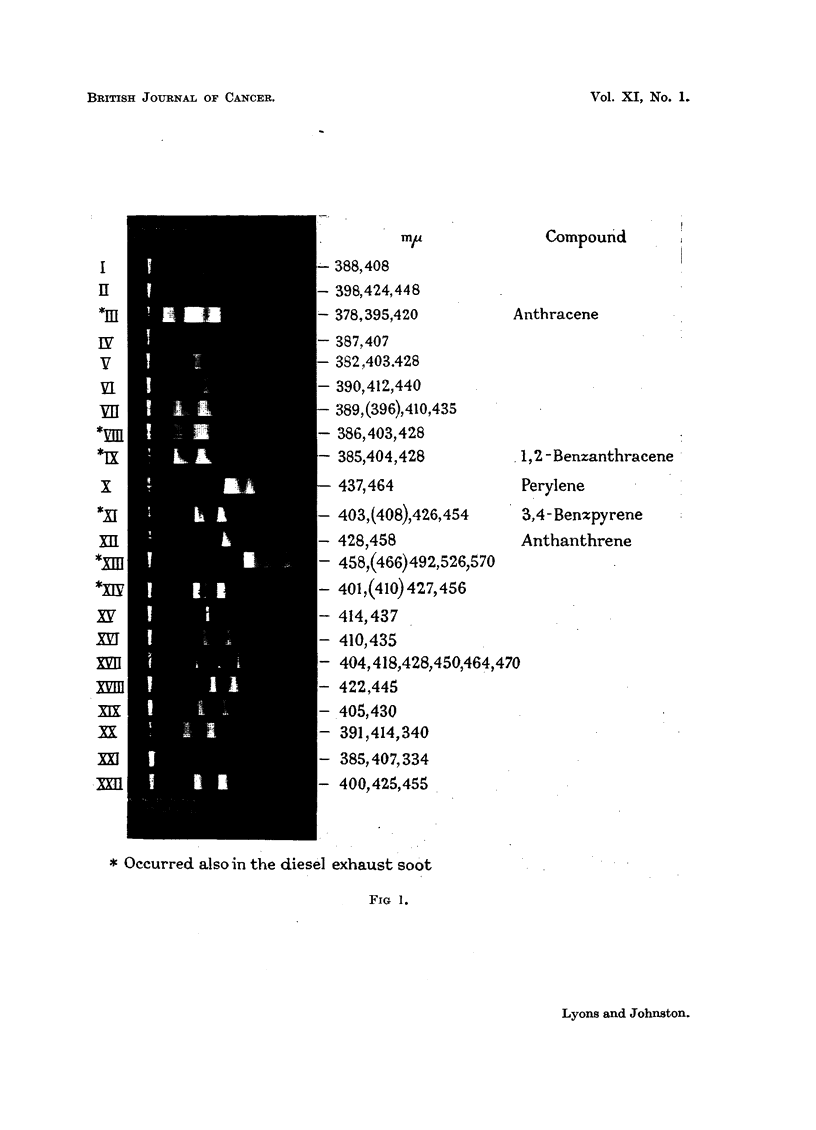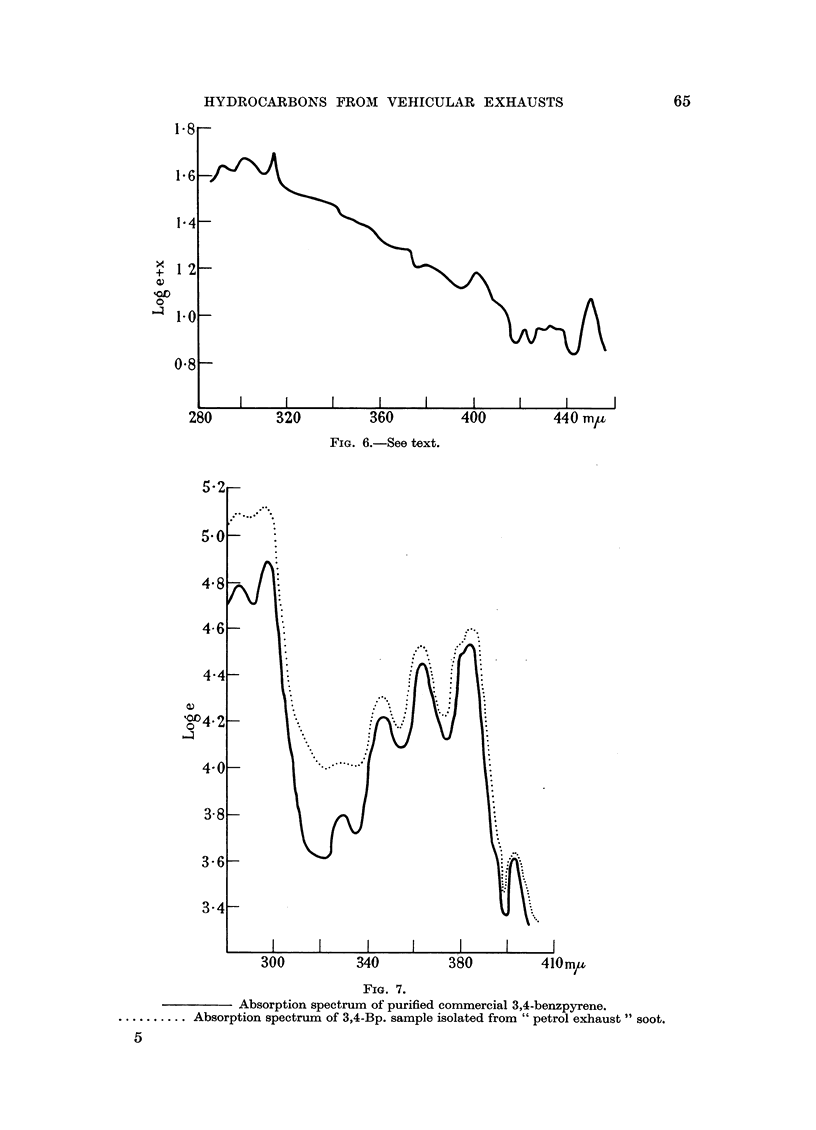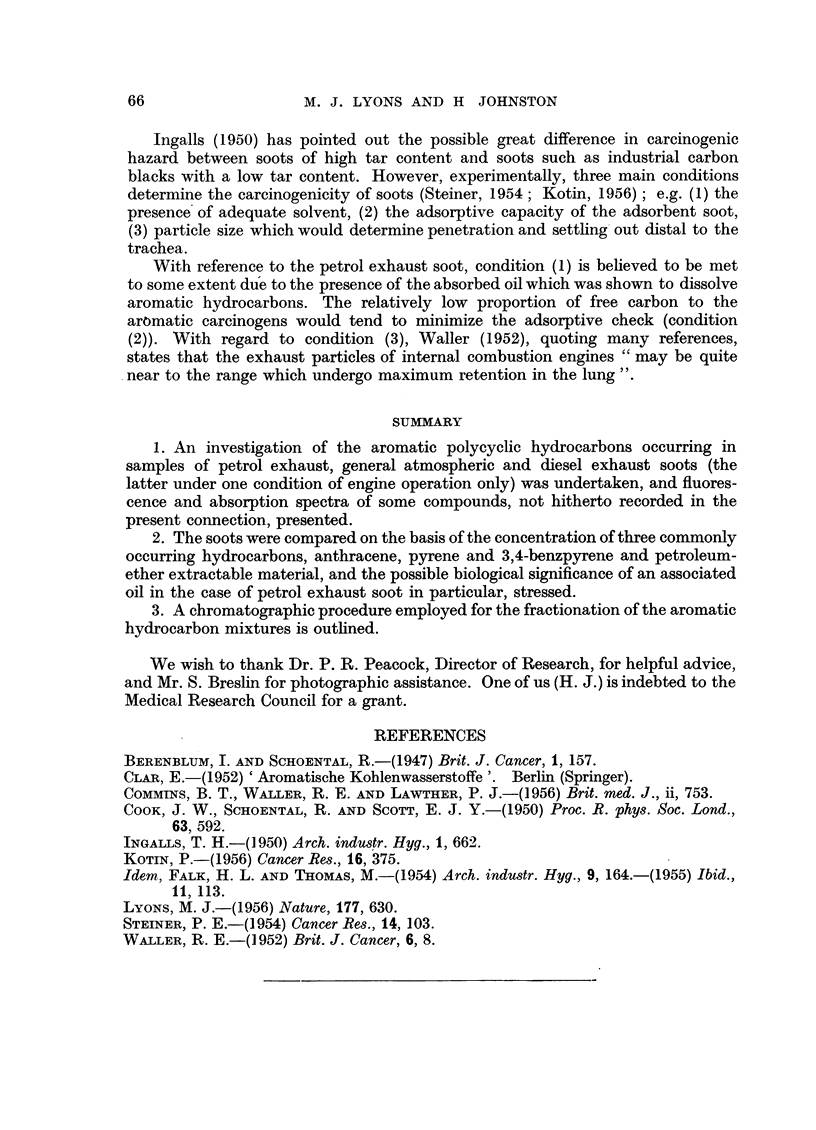# Aromatic Hydrocarbons from Vehicular Exhausts

**DOI:** 10.1038/bjc.1957.9

**Published:** 1957-03

**Authors:** M. J. Lyons, H. Johnston

## Abstract

**Images:**


### AROMATIC HYDROCARBONS FROM VEHICULAR EXHAUSTS

M. J. LYONS AND H. JOHNSTON
*From the Cancer Research Department, Royal Beatson Memorial Hospital, Glasgow*

Received for publication December 21, 1956

THE greater frequency of cancer of the lung in urban over rural populations has been well established. This has prompted the evaluation of the aetiological relevancy of agents which have been introduced or which have increased in quantity in urban atmosphares in recent years. Thus, Kotin, Falk and Thomas (1954, 1955), sampling the particulate phase of exhausts from both petrol and diesel engines, identified seven polycyclic hydrocarbons spectroscopically, including the traditional animal carcinogen, 3, 4-benzpyrene, and showed their differential formation with respect to engine speed and load.

The present paper records an assay for aromatic hydrocarbons of a sample of petrol exhaust soot produced under the varying conditions of normal automobile running and compares the results with those obtained from a sample of general atmospheric soot. A sample of diesel exhaust soot, collected as the engine idled, was similarly investigated.

### EXPERIMENTAL

The twin exhaust pipes of a 3·6-litre private car were muzzled with fine hemp sacks and an exhaust sample collected over a short period of normal motoring. The sacks and contents were extracted by refluxing with acetone. The acetone extract was taken to dryness and the residue extracted by refluxing for three 3-hour periods with petroleum-ether (B.P. 60–80° C.). The combined extracts were taken to a small volume, and the free carbon of the residual soot estimated. The concentrated extract was fractionated by adsorption chromatography.

The following chromatographic procedure was employed. The concentrated extract, which contained 4·56 g. of extracted material, was layered on top of a 30 × 4 cm. column of alumina (100–200 mesh) and developed with 60–80° petroleum-ether. The progress of the fractionation in this and subsequent chromatograms, was followed by periodic inspection of the columns under a filtered U.V. lamp. All columns were screened from diffuse daylight by wrappings of black paper, to minimize incidental photo-oxidation.

In the initial chromatogram, a fast moving slightly fluorescent oil was detected in the eluate. For the full development of the chromatogram, chloroform was incorporated in the eluent to a concentration of 40 per cent. Thus more strongly adsorbed components were eluted. In all, twelve fractions were collected.

As considerable overlapping of zones occurred, each fraction was separately chromatographed on 20 × 2·5 cm. columns of 100–200 mesh alumina (Spence). The first three fractions were chromatographed using petroleum-ether as eluent. The remaining nine fractions were each developed with petroleum ether containing

2 per cent to 8 per cent acetone. Of various solvent mixtures tried, petroleum-ether containing low concentrations of acetone had been shown to be most suitable for the separation of higher polycyclic hydrocarbons. Discrete zoning is achieved and development is very rapid. The various sub-fractions were purified by rechromatography on 100–200 mesh Silica Gel (Light & Co.) with petroleum-ether as solvent, screened ((*a*) by fluorescence spectrography using a Hilger E.3 quartz spectrograph and excitation radiation 3650 A ; (*b*) by taking absorption spectra in the range 260 m$\mu$–460 m$\mu$. Fractions showing similar spectra were combined and rechromatographed on alumina and silica gel as described above.

Fig. 1 records the fluorescence spectra of compounds separated in the above manner, in approximate order of decreasing solubility. Exposures varied from 3 to 15 minutes. Ilford H.P.3 plates were used initially with ID-11 as developer. The spectra on Fig. 1 were so prepared. However, Ilford " Lond Range Spectrum " plates have been found to give more sharply defined spectra.

According to the relationship noted by Cook, Schoental and Scott (1950), compounds X (Perylene), XII (anthanthrene) and XIII (which has a brilliant green fluorescence) have a minimum of two quinonoid rings, the rest of the compounds having a minimum of one. Compounds XX and XXI (Fig. 1) resemble spectrographically compounds isolated from horizontal retort tar by Berenblum and Schoental (1947), who showed that two fractions which recorded main fluorescence bands at 391 m$\mu$ and 385 m$\mu$ respectively, were carcinogenic for rabbit skin.

U.V. absorption spectrophotometry confirmed the presence of the compounds identified by the spectrographic method. In addition, the following compounds were identified : pyrene, " Compound X " (Kotin, 1954), 1,2-benzpyrene, 1,12-benzperylene, and coronene. Fig. 2–6 represent the absorption spectra of compounds isolated from the petrol exhaust soot, all of which were more strongly adsorbed on alumina than 3,4-benzpyrene. Identification was rendered difficult as the quantities involved were small and contaminants difficult to separate.

Fig. 2 represents a compound with a brilliant blue-violet fluorescence which occurs in diesel soot, atmospheric soot, and cigarette smoke (Lyons, 1956, Fluorescent Spectrum IV) as well as petrol soot. It gives a characteristic fluorescence spectrum (XIV). It has the following absorption maxima in cyclohexane : 401, 380, 360, 308, 296, 282 m$\mu$.

Fig. 3. This fraction had a violet fluorescence. The fluorescence spectrum is presented (XX). Its absorption spectrum in cyclohexane had the following maxima : 391, 383, 366, 348, 331, 306, 290 m$\mu$. The spectrum resembles that of 3,4-benztetraphene (Clar, 1952, p. 209).

Fig. 4 and 5 both have blue-violet fluorescence and are closely associated chromatographically. Absorption maxima, with benzene as solvent, were : Fig. 4 : 408, 405, 396, 376, 356, 340; 308, 296, 284 m$\mu$. Fig. 5 : 420, 405, 396, 368, 346, 328 (308), 304, 296 m$\mu$.

Fig. 6. This spectrum was given by a fraction which had a blue fluorescence. It is obviously a mixture of two or more compounds. They occurred in too low a concentration to achieve an effective separation. The presence of the potent carcinogen, 3,4,8,9-dibenzpyrene is suggested (Clar, 1952, p. 345). Absorption maxima in benzene were : 452, 424, 402, 380, 314, 300, 290 m$\mu$.

Fig. 7 represents the Log *e* absorption spectrum of a sample of the 3,4-benzpyrene isolated from petrol soot, with a standard sample for comparison.

In the chromatography of both the petrol and diesel soots the aromatic hydrocarbons were preceded by an oil which was weighed following removal of the solvent. This oil was considered to be unburnt fuel and overhead lubricant. It readily dissolved crystalline 3,4-benzpyrene and other aromatic hydrocarbons.

Some of the major occurring hydrocarbons in petrol, diesel and atmospheric soot were estimated by the base-line technique. The results are presented in Table I with reference to parts per million of free carbon. Also included in Table I are the ratios of acetone extracted material, petroleum-ether extract and oil to free carbon.

TABLE I.

| | Atmospheric soot | Petrol exhaust soot | Diesel exhaust soot |
|---|---|---|---|
| 3,4-benzpyrene (p.p.m. free carbon) | 380 | 1570 | 20 |
| Pyrene (p.p.m. carbon) | 650 | 440 | 820 |
| Anthracene (p.p.m. carbon) | 215 | 385 | 60 |
| 1,2-benzanthracene (p.p.m. carbon) | — | 180 | — |
| Acetone extract/free carbon | 1·06 | 3·50 | 0·6 |
| Pet.-ether extract/free carbon | 0·17 | 3·49 | 0·58 |
| Oil/free carbon | — | 0·92 | 0·29 |

### DISCUSSION

The presence and intensity of absorption maxima, and fluorescence spectra where the requisite reference compounds were available, were the only means employed to identify the aromatic hydrocarbons separated from the various soots. Fluorescence spectrography proved valuable in segregating the various compounds, but sometimes superimposition rendered the interpretation of the spectra difficult. Quantitative differences occurred in the relative concentrations of the hydrocarbons. Chrysene was the only aromatic hydrocarbon occurring in the atmospheric soot which was not detected in the vehicular exhaust soots. On the other hand the brilliant green fluorescent compound (Fig. 1, XII) which is being further purified, occurring in reasonable concentration in the latter, was not detected in the atmospheric soot. The compound (Fig. 1, XIV) found in all three soot samples was relatively diluted in the atmospheric soot. Thus, if vehicular exhausts were shown to be the chief source of emission of this compound into the atmosphere, it is conceivable that its index of dilution in atmospheric soot could be used to gauge the atmospheric pollution component due to vehicular exhausts.

The demonstration of 3,4-benzpyrene as the hydrocarbon of highest concentration of those estimated in the sample of petrol soot is at variance with the data of Kotin, Falk and Thomas (1954), which would place pyrene at higher concentration to 3,4-benzpyrene. The low concentration of 3,4-benzpyrene in the diesel soot, sampled as the engine idled, conforms to the concentrations achieved by Commins, Waller and Lawther (1956).

On comparison (Table I) of the petrol exhaust soot with the general atmospheric soot two significant differences were noted, apart from the possible formation of a highly potent carcinogen like 3,4,8,9-dibenzpyrene and compounds XX and XXI in the petrol internal combustion engine :

(*a*) The relatively high proportion of 3,4-benzpyrene in petrol exhaust soot ; (*b*) the relatively high ratio of petroleum-ether extract to free carbon in petrol exhaust soot, in which was reflected the presence of a considerable quantity of oil (26·4 per cent in petroleum ether extract).

FIG. 2.—See text.

FIG. 3.—See text.

FIG. 4.—See text.

FIG. 5.—See text.

---

EXPLANATION OF PLATE.

FIG. 1.—Fluorescence spectra of compounds isolated from " petrol exhaust " soot. Solvent, cyclohexane.

mμ

Compound

I — 388,408

II — 398,424,448

*III — 378,395,420 Anthracene

IV — 387,407

V — 382,403,428

VI — 390,412,440

VII — 389,(396),410,435

*VIII — 386,403,428

*IX — 385,404,428 1,2-Benzanthracene

X — 437,464 Perylene

*XI — 403,(408),426,454 3,4-Benzpyrene

XII — 428,458 Anthanthrene

*XIII — 458,(466)492,526,570

*XIV — 401,(410) 427,456

XV — 414,437

XVI — 410,435

XVII — 404,418,428,450,464,470

XVIII — 422,445

XIX — 405,430

XX — 391,414,340

XXI — 385,407,334

XXII — 400,425,455

* Occurred also in the diesel exhaust soot

FIG 1.

FIG. 6.—See text.

FIG. 7.

———— Absorption spectrum of purified commercial 3,4-benzpyrene.

.......... Absorption spectrum of 3,4-Bp. sample isolated from " petrol exhaust " soot.

Ingalls (1950) has pointed out the possible great difference in carcinogenic hazard between soots of high tar content and soots such as industrial carbon blacks with a low tar content. However, experimentally, three main conditions determine the carcinogenicity of soots (Steiner, 1954 ; Kotin, 1956) ; e.g. (1) the presence of adequate solvent, (2) the adsorptive capacity of the adsorbent soot, (3) particle size which would determine penetration and settling out distal to the trachea.

With reference to the petrol exhaust soot, condition (1) is believed to be met to some extent due to the presence of the absorbed oil which was shown to dissolve aromatic hydrocarbons. The relatively low proportion of free carbon to the arömatic carcinogens would tend to minimize the adsorptive check (condition (2)). With regard to condition (3), Waller (1952), quoting many references, states that the exhaust particles of internal combustion engines " may be quite near to the range which undergo maximum retention in the lung ".

### SUMMARY

1. An investigation of the aromatic polycyclic hydrocarbons occurring in samples of petrol exhaust, general atmospheric and diesel exhaust soots (the latter under one condition of engine operation only) was undertaken, and fluorescence and absorption spectra of some compounds, not hitherto recorded in the present connection, presented.

2. The soots were compared on the basis of the concentration of three commonly occurring hydrocarbons, anthracene, pyrene and 3,4-benzpyrene and petroleum-ether extractable material, and the possible biological significance of an associated oil in the case of petrol exhaust soot in particular, stressed.

3. A chromatographic procedure employed for the fractionation of the aromatic hydrocarbon mixtures is outlined.

We wish to thank Dr. P. R. Peacock, Director of Research, for helpful advice, and Mr. S. Breslin for photographic assistance. One of us (H. J.) is indebted to the Medical Research Council for a grant.

### REFERENCES

Berenblum, I. and Schoental, R.—(1947) *Brit. J. Cancer*, **1**, 157.
Clar, E.—(1952) ' Aromatische Kohlenwasserstoffe '. Berlin (Springer).
Commins, B. T., Waller, R. E. and Lawther, P. J.—(1956) *Brit. med. J.*, ii, 753.
Cook, J. W., Schoental, R. and Scott, E. J. Y.—(1950) *Proc. R. phys. Soc. Lond.*, **63**, 592.
Ingalls, T. H.—(1950) *Arch. industr. Hyg.*, **1**, 662.
Kotin, P.—(1956) *Cancer Res.*, **16**, 375.
*Idem*, Falk, H. L. and Thomas, M.—(1954) *Arch. industr. Hyg.*, **9**, 164.—(1955) *Ibid.*, **11**, 113.
Lyons, M. J.—(1956) *Nature*, **177**, 630.
Steiner, P. E.—(1954) *Cancer Res.*, **14**, 103.
Waller, R. E.—(1952) *Brit. J. Cancer*, **6**, 8.